# Nocturnal pruritus and sleep disturbance associated with dermatologic disorders in adult patients

**DOI:** 10.1016/j.ijwd.2021.02.010

**Published:** 2021-03-03

**Authors:** Indrashis Podder, Himel Mondal, George Kroumpouzos

**Affiliations:** a Department of Dermatology, Venereology, and Leprosy, College of Medicine and Sagore Dutta Hospital, Kolkata, West Bengal, India; b Department of Physiology, Bhima Bhoi Medical College and Hospital, Balangir, Odisha, India; c Department of Dermatology, Alpert Medical School of Brown University, Providence, Rhode Island; d Department of Dermatology, Medical School of Jundiaí, São Paulo, Brazil; e GK Dermatology, PC, South Weymouth, Massachusetts

**Keywords:** Nocturnal pruritus, sleep disturbance, skin diseases, quality of life, therapy

## Abstract

Nocturnal pruritus (NP) is a relatively common reason for dermatologic consultation. Its pathophysiology is partially understood. Skin conditions such as atopic dermatitis, psoriasis, urticaria, and prurigo nodularis are well-described causes of NP. The most distressing sequela of NP is sleep deficit, which can lead to physical and mental disturbances (e.g., daytime somnolence and fatigue) and negative emotional states that profoundly affect quality of life. However, this aspect of NP is often overlooked by dermatologists. It is essential to assess sleep quality in such patients and adopt appropriate measures to arrest the problem at an early stage. We conducted an evidence-based literature review to highlight the pathogenetic mechanisms of NP, identify dermatologic etiologies, and explore methods that have been used to assess the quality of sleep. Furthermore, we performed a systematic review of studies on sleep disturbance relevant to NP in patients with dermatologic conditions. Finally, we discuss the evidence on treatment options for NP and indicate therapies that may target both NP and sleep disturbance.

## Introduction

Pruritus commonly exhibits nocturnal exacerbation, disturbing normal sleep patterns and quality (Tivoli and Rubenstein, 2009). Sleep is an active process coupled with the circadian rhythm (CR) that is essential for optimal physical and mental health. Pathophysiologic mechanisms, such as skin barrier dysfunction and altered serum levels of endogenous substances (e.g., cortisol and signaling molecules), may be responsible for NP (Lavery et al., 2016). NP is a common dermatologic complaint, typically associated with sleep deficits and impaired quality of life. However, sleep disturbance (SD) is often overlooked in the current assessment instruments of quality of life in skin disorders, which results in a practice gap. Unfortunately, most studies in dermatologic conditions have evaluated sleep as a secondary outcome using subjective methods.

## Physiology of sleep

**Fig. 1:**
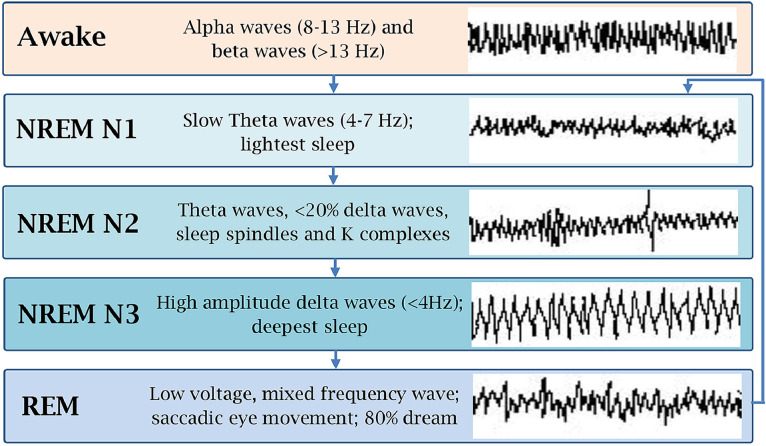
Stages of sleep with respective electroencephalogram findings.

A person passes from wakefulness to sleep when a network of cortical structures that maintain cortex activation is suppressed by inhibitory neurons. A high concentration of adenosine has been suggested to play an important role in such inhibition (Potter et al., 2016). Behavioral and physiologic monitoring has divided the sleep cycle into two distinct stages with distinct physiochemical changes: nonrapid eye movement (NREM) sleep and rapid eye movement (REM) sleep. When a person enters from wakefulness to sleep, they first enter the NREM stage (75%-80% of sleep) and then the REM stage (20%-25% of sleep; Fig. 1). This is repeated cyclically, with each cycle lasting 90 to 110 minutes and 4 to 6 cycles occurring per night (Carley and Farabi, 2016).

The sleep–wake cycle is interwoven with the CR of the body. The hypothalamic suprachiasmatic nucleus plays a key role in controlling the CR, resulting in diurnal and nocturnal variations (Carley and Farabi, 2016). Any disruption of the CR may cause behavioral (e.g., eating and physical activity) and biological (e.g., renal dysfunction) imbalances. Lifestyle (e.g., duty in night shift) and environmental factors (e.g., bright lights during the nighttime) can disrupt the CR, thus hampering sleep hygiene (Folkard, 2008; Potter et al., 2016). Excessive daytime sleepiness, mood disturbance, and insomnia are some notable sequelae of disturbed sleep. Several metabolic functions may also be deranged by sleep deprivation, which increases the risks of diabetes mellitus, sympathetic nervous system hyperactivity, hypertension, ischemic heart disease, and impaired immunity (Parish, 2009).

NP exerts a detrimental effect on the sleep pattern. Arousal does not occur prior to itching, but scratching behavior ensues (Lavery et al., 2016). Itching is more prevalent in stage N1 but may also occur in stage N2 of NREM and REM (Fig. 1; Monti et al., 1989). The deeper, slow-wave, N3 sleep stage is the least affected by NP, possibly due to reduced sensory perception (Lavery et al., 2016).

## Pathophysiology of nocturnal pruritus

Several key functions of the skin may be altered during sleep, including thermoregulation, maintenance of fluid balance, and barrier function (Lavery et al., 2016). Aberrations in such regulatory mechanisms can contribute to NP. Additionally, hormonal variations may be involved (Patel et al., 2007).

### Thermoregulation variation

Along with playing a major role in maintaining the CR, the hypothalamus changes the core body temperature (Gupta and Gupta, 2013). It is highest in late evening and lowest in early morning. The core body temperature is also reduced in the NREM stages of sleep. Hence, the skin becomes hot while dissipating heat to the environment, and this heat can increase intensity of pruritus, resulting in NP (Gupta and Gupta, 2013).

### Barrier function and fluid balance alteration

The stratum corneum acts as a physical barrier to allergens and pruritogens. Therefore, a defective barrier function may exacerbate NP. The barrier function is assessed by estimating transepidermal water loss (TEWL). TEWL increases during the night and decreases in the early morning. Therefore, barrier function decreases during the night, which may allow entry of pruritogens to stimulate itching. Notably, higher TEWL has been reported in patients with atopic dermatitis (AD; Gupta et al., 2016; Patel et al., 2007).

### Hormonal dysregulation

Cortisol secretion parallels the CR. Its concentration is lower during the evening and at midnight and gradually builds up overnight to peak in the early morning. As the concentration of corticosteroids is at a nadir during the evening and night, their anti-inflammatory effects reflect this pattern, possibly resulting in increased nocturnal itching. The concentration of melatonin increases during the night and subsequently decreases during the daytime to baseline level. In patients with AD, melatonin levels decrease during the night (Lavery et al., 2016). Reduced melatonin levels at nighttime may contribute to NP (Chang and Chiang, 2016). Also, melatonin levels may be reduced when normal nighttime sleep is disturbed by pruritus (Gupta et al., 2016).

### Cytokine involvement

Interleukin (IL) 1β and tumor necrosis factor-alpha regulate NREM sleep. SD can increase the level of plasma proinflammatory cytokines IL-1β and tumor necrosis factor-alpha. These may cause increased itching by stimulating the inflammatory cascade of dermatitis. The level of IL-2, a pruritogenic cytokine, increases at night and may result in NP (Lavery et al., 2016).

### Itching and pain

A reduction in pain can induce itching. The μ-opioid receptor agonists and κ-opioid receptor antagonists can induce itching. The dysfunctional release of different opioids may induce NP (Lavery et al., 2016; Patel et al., 2007).

### Itching and psyche

Stress alters the thermoregulation and hemodynamic balance of the body and subsequently elevates the serum levels of histamine and other endogenous pruritogens. Also, stress may impair the hypothalamo–pituitary–adrenal axis, leading to reduced serum cortisol concentration, as reported in patients with AD (Schut et al., 2016). Depression can lower the central itch threshold by increasing opiate levels and disturbing the balance between the µ- and κ-opioid receptor pathways (Jafferany and Davari, 2019).

## Methods that assess sleep quality

Subjective and objective methods are listed in Table 1. Although objective methods are more accurate, they are difficult to implement and may be reserved for more severe cases. Subjective methods are multi-item scales that assess the quality of sleep and are easier to apply on an outpatient basis. However, none of these scales have been validated in chronic pruritus (CP; persistent itch >6 weeks; Suilmann et al., 2018). The Pittsburg Sleep Quality Index is one of the most widely used indices in clinical studies and has been used most commonly in AD and psoriasis (Kaaz et al., 2019). Interestingly, some authors found the Regensburg Insomnia Scale to be most suitable for use in CP (Suilmann et al., 2018). Questionnaires specifically developed for AD have been validated (Lei et al., 2020a; 2020b).

**Table 1:** Methods that assess sleep quality

Subjective	Objective
• Stanford sleepiness scale (Hoddes et al., 1973): One-item scale that assesses alertness on the following day • Epworth sleepiness scale (Johns, 1991): Eight-item scale that estimates the risk of falling asleep in eight daily situations • Medical outcomes study sleep scale (Allen et al., 2009): 12-item scale that also addresses respiratory impairment • Pittsburg sleep quality index (Buysse et al., 1989:; 24-item scale that assesses sleep quality retrospectively over the last 4 weeks • Athens insomnia scale (Soldatos et al., 2000): Eight-item scale that assesses daytime sleepiness • Glasgow sleep effort scale (Broomfield and Espie, 2005): Seven-item scale that assesses effort to fall asleep • Regensburg insomnia scale (Cronlein et al., 2013): Ten-item-scale that assesses quantitative aspects of sleep	• **Polysomnography** (Togeiro and Smith, 2005): Criterion standard method; consists of electroencephalogram, electooculogram, and electromyogram; requires a sleep center • **Actigraphy** (Bender et al., 2003; Jeon et al., 2017; Togeiro and Smith, 2005): Wrist-worn device monitors sleep activity and records awakenings; although less accurate than polysomnography, this method is easier, cost effective, and deemed suitable for dermatological disorders

## Methods and materials

**Fig. 2:**
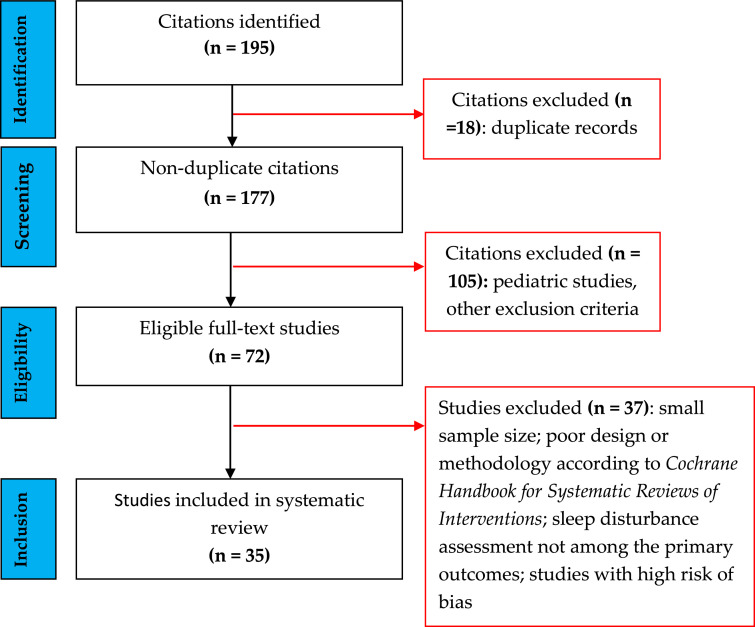
Flow diagram of literature search and selection of studies.

We conducted an evidence-based, systematic review on the prevalence and types of SD in adult patients with dermatologic disorders per the Preferred Reporting Items for Systematic Reviews and Meta-analysis guidelines. Figure 2 depicts the study selection. We searched for articles in the English language in the MEDLINE, Scopus, and Cochrane databases using the following search items: *NP* OR *nocturnal itching* AND *impaired quality of sleep* OR *SD* AND *dermatology* OR *skin* OR *cutaneous* OR *psychocutaneous*. Randomized controlled trials, case-control studies, and cohort studies were included. Studies performed in children and adolescents, animal studies, reviews, expert opinions, and book chapters were excluded. Additionally, the reference lists of selected or review articles were screened for additional studies. We evaluated the quality of the studies and assessed their bias using the *Cochrane Handbook for Systematic Reviews of Interventions*. Studies with a high risk of bias were excluded, as were those in which SD was not among the primary outcomes. All authors reviewed the eligible articles, and disagreements among the authors were discussed until a consensus was reached.

## Results

A total of 35 studies were obtained from the systematic review (Table 2).

**Table 2:** Dermatologic conditions associated with NP and SD

Dermatologic disorder (studies)	Prevalence and major SD types	Possible mechanisms of SD	Comments
**Atopic dermatitis** (Ebata et al., 1999; Kim et al., 2020; Kong et al., 2016; Li et al., 2018; Silverberg et al., 2015; 2018a; 2018b; 2020; Simpson et al., 2016; 2018; Vakharia et al., 2017; Wei et al., 2018; Yano et al., 2013; Yu et al., 2016)	SD in 33%-90% of pts; ↓ SQ, ↓ sleep efficiency*, ↑ SL†, premature sleep awakening; ↑ total scratching time at night, correlates to disease severity; scratching occurs mainly in N1 and N2 stages; ↑ daytime fatigue, ↓ activity of daily living/QoL	CP in 87%-100% of pts; NP and scratching are the most important determinants of SD; epidermal barrier dysfunction can facilitate entry of pruritogens; immune system-mediated release of pruritogenic cytokines; nighttime changes in skin physiology and cytokines can exacerbate atopic inflammation, itch, SD	Severity of AD correlates with SD (↑ total scratching time at night correlates to disease severity); SD and AD may be intricately related (vicious cycle); degree of SD correlates with ↓ QoL; dupilumab treatment decreases SD (Cork et al., 2020; Fargnoli et al., 2019, Tsianakas et al., 2018)
**Psoriasis** (Hawro et al. 2020; Henry et al., 2017; Jensen et al., 2018; Kimball et al., 2016; Ljossa et al., 2012; Shutty et al., 2013; Smith et al. 2019; Tas et al., 2020; Wu at al., 2008; Zachariae et al., 2012)	SD in 39%-82% of pts; ↓ SQ, ↑ SL, ↓ sleep duration/efficiency, ↑ premature sleep awakening, trouble sleeping due to being too hot and experiencing pain; ↑ daytime somnolence; ↓ QoL	Disorder of thermoregulation and heat loss mechanism leading to ↑ CBT (↓ CBT necessary for sleep initiation); ↓ itch threshold leading to NP; multiple factors affect sleep including pruritus, disease severity, obesity, OSAHS, smoking, comorbid conditions (especially depression), cognitive and somatic arousal	SD showed positive correlation with itch and PASI scores; etanercept (Mrowietz et al., 2015), adalimumab (Strober et al., 2012), and betamethasone dipropionate/calcipotriol (Jalili et al., 2019; Kontochristopoulos et al., 2016) treatments ↑ SQ; psoriasis not a/w SD in 1 study (Stinco et al., 2013)
**Hidradenitis suppurativa** (Kaaz et al., 2018; Vossen at al., 2017)	SD in 70.4% of pts; ↓ sleep duration, ↑ SL, habitual sleep efficiency; daytime dysfunction	Itch and pain a/w SD; activation of Th17 and Th2 pathways serum level of pruritogenic cytokines; itch and pain have an important impact on insomnia and SQ	Often a/w anxiety, fatigue, depression and stigmatization; pain is a crucial factor in SD
**Chronic spontaneous urticaria** (Abdel, 2017)	SD in 48.8% of pts; ↑ sleep latency, ↓ sleep duration, ↑ daytime somnolence	Release of histamine, substance P, and CGRP, which ↑ NP and affect SQ	Urticaria severity score – 7 items is proportional to degree of SD; omalizumab treatment may improve SQ (Gimenez et al., 2016)
**Infestations (e.g., scabies)** (Worth et al., 2012)	SD in 87.5% of pts; ↓ SQ, ↓ sleep duration, ↑ premature awakenings	Scabies mites more active at night; feces (scybala) activate protease activating receptor-2 (pruritic receptor); Th2 immunity against mite results in production of potent pruritogens, such as IL-31	Only cause of NP with acute onset and short duration; SD improves promptly with ivermectin treatment

a/w, associated with; CBT, core body temperature; CGRP, calcitonin gene related peptide; CP, chronic pruritus; IL, interleukin; NP, nocturnal pruritus; OSAHS, obstructive sleep apnea hypopnea syndrome; PASI, psoriasis area severity index; pts, patients; QoL, quality of life; SD, sleep disturbance; SL, sleep latency; SQ, sleep quality; Th, T helper cell

⁎ Sleep efficiency refers to the proportion of time in bed spent sleeping.

† SL refers to the time between going to be asleep and sleep onset.

### Dermatologic conditions associated with nocturnal pruritus and sleep disturbance

The dermatologic conditions association with NP and SD are detailed in Table 2. Most of these disorders manifest with CP, and NP has been reported in almost 90% of patients with CP (Lavery et al., 2017). The severity of NP correlated with the severity of SD and overall itching in a CP population (Lavery et al., 2017). Psychocutaneous conditions have also been associated with NP and SD; however, there are scarce data (Gupta and Gupta, 2013; Kuhn et al., 2017a; 2017b). Similarly, there have been only small series on prurigo nodularis and lichen simplex chronicus (Gwillim et al., 2020; Koca et al., 2006). In a recent study, SD was more common in patients with skin diseases compared with healthy controls (Shen et al., 2020). AD and rosacea were the most common associated skin disorders in the study. Systemic inflammation has been suggested as a possible link between chronic pruritic dermatoses and SD because an increased serum C-reactive protein level was documented in patients with SD (Patel et al., 2021).

### Treatment of nocturnal pruritus

There is a scarcity of controlled data on NP therapy. However, many treatment options for CP can also improve NP (Lavery et al., 2016; Table 3). There are very few SD intervention trials in patients with skin disorders (Bawany et al., 2020). Treatment of the skin disorder itself with biologic (i.e., dupilumab for AD, and adalimumab or etanercept for psoriasis) or topical agents can have beneficial effects on both NP and SD (Table 2). Therapy for NP includes nonpharmacologic and pharmacologic treatments.

**Table 3:** Therapeutic options for nocturnal pruritus with respective level of evidence and grade of recommendation

Treatment	Level of evidence*	Grade of recommendation
Nonpharmacologic		
Emollients and wet-wrap therapy (González-López et al., 2017; Pereira and Ständer, 2017) Coolants (Kuhn et al., 2017b) Address bathing habits (Haghayegh et al., 2019) Sleep hygiene (Chang and Chiang, 2016) Psychotherapeutic interventions (Bae et al., 2012; Jafferany and Davari, 2019; Schut et al., 2016)	2b 2b 1a 2b 2b	B B A B B
Pharmacologic-topical (Kontochristopoulos et al., 2016; Pereira and Ständer, 2017; Kuhn et al., 2017b)		
Anesthetics Corticosteroids Calcineurin inhibitors Miscellaneous agents (naltrexone, polidocanol, aprepitant)	1b 1b 4 5	A A C D
Pharmacologic – systemic		
H1 antihistamines (Lavery et al., 2016; Yamanaka et al, 2015)Antidepressants (Khanna et al., 2019; Kuhn et al., 2017b; Lavery et al., 2016; Savin et al., 1979; Yosipovitch and Samuel, 2008) ○ Mirtazapine ○ Doxepine Gamma-aminobutyric acid agonists (Lavery et al., 2016; Pereira and Ständer, 2017)Opioid receptor modulators (Kozono et al., 2018; Dawn and Yosipovitch, 2006)Benzodiazepines (Ebata et al.,1998; Roux and Kryger, 2010)Melatonin (Chang et al., 2016)Biologics (dupilumab, adalimumab, etanercept; Cork et al., 2020; Fargnoli et al., 2019; Mrowietz et al., 2015; Strober et al., 2012; Tsianakas et al., 2018)Phototherapy (Becker et al., 2011; Kuhn et al., 2017b; Yosipovitch and Bernhard, 2013)	1a 1b 2b 1b 1b 4 5 2b 2b	A A B A A C D B B

⁎ Level of evidence and grade of recommendation were assigned according to validated scale developed by Oxford Centre for Evidence-based Medicine (Burns et al., 2011).

### Nonpharmacologic treatments

Nonpharmacologic treatments may be adopted as first-line therapy because they are safe, easy to implement, and cost effective. These treatments may be supplemented by pharmacologic therapy in cases of inadequate response. The authors of the reviewed articles recommend emollients and coolants, addressing bathing habits and adequate sleep hygiene in all patients. Furthermore, appropriate psychologic interventions may be adopted in selected patients after proper counseling.

#### Emollients and coolants

Emollients should be recommended to all patients with NP, especially because they have been established in CP (Pereira and Ständer, 2017). By improving the epidermal barrier function and reducing TEWL (Yosipovitch and Bernhard, 2013), they help alleviate NP. However, their efficacy in NP has not been evaluated in randomized controlled trials. These agents should be applied at bedtime, preferably on moist skin. Nocturnal wet-wrap application (i.e., application of emollients under wet gauze or night-suit) can enhance the efficacy of emollients, as demonstrated in AD (González-López et al., 2017). Coolants, such as menthol (1%-5%), stimulate κ-opioid receptors and thermosensitive ion channels, thereby producing a cool sensation that helps alleviate pruritus (Kuhn et al., 2017b). This is especially important for NP exacerbated by heat.

#### Addressing bathing habits

A systematic review reported that taking a warm-water (40-42.5°C) bath/shower for 10 minutes 1 to 2 hours before bedtime improved sleep quality and efficiency and significantly reduced sleep latency (Haghayegh et al., 2019). A suggested mechanism involves increased blood perfusion to the palms and soles, thus augmenting the distal-to-proximal skin temperature gradient and facilitating body heat dissipation. As the skin loses heat, it becomes cooler, which may reduce the intensity of NP.

#### Sleep hygiene

Sleep hygiene refers to having a bedroom environment and daily routine that promote uninterrupted sleep. Sleep hygiene plays a role in reducing NP. Blue light from electronic screens is known to suppress melatonin secretion, resulting in impaired sleep quality (Chang and Chiang, 2016). Thus, a dark sleep environment is recommended, and light produced by mobile and computer screens should be eliminated.

#### Psychotherapeutic interventions

In disturbed sleep due to NP that is unresponsive to the former measures, psychotherapeutic interventions, such as relaxation techniques, habit-reversal training, rational emotive therapy, and cognitive behavioral therapy, can be tried, especially because stress and anxiety are usually involved (Jafferany and Davari, 2019; Schut et al., 2016). Progressive muscle relaxation has been helpful in reducing CP and loss of sleep in AD (Bae et al., 2012). Patients’ understanding about the disease and associated aggravating/relieving factors and their perception is of paramount importance (Jafferany and Davari, 2019).

### Pharmacologic treatments

Systemic agents are the mainstay of therapy; however, topical therapies may provide additional benefit.

### Topical therapies

#### Anesthetics

Capsaicin (0.025%-0.1%), pramoxine (1%-2.5%), and a mixture of lidocaine (2.5%-5%) and prilocaine (2.5%) have been used alone or in combination to obtain relief from CP (Kuhn et al., 2017b). These agents are especially beneficial in neuropathic itching because their primary mode of action involves the desensitization of the peripheral nerves and depleting substance P, a known pruritogen.

#### Corticosteroids

Although topical corticosteroids are essentially anti-inflammatory rather than antipruritic agents, they have been beneficial for pruritus and SD in adults with inflammatory conditions such as psoriasis (Kontochristopoulos et al., 2016). Their therapeutic role may be explained by counteracting the nighttime reduction of serum cortisol (Chang and Chiang, 2016).

#### Calcineurin inhibitors

Although studies on calcineurin inhibitor (i.e., tacrolimus, pimecrolimus) use in NP are lacking, their use for NP associated with inflammatory skin diseases, such as AD and psoriasis, may be attempted based on experience with CP (Pereira and Ständer, 2017).

#### Miscellaneous agents

Naltrexone (1% cream), polidocanol (3% cream), and aprepitant cream have been beneficial in treating pruritic skin disorders, such as AD, psoriasis, prurigo nodularis, and lichen simplex chronicus (Kuhn et al., 2017b).

### Systemic therapies

#### H1 antihistamines

Antihistamines are first-line therapy for managing NP. However, their use in NP as monotherapy is inadequate because NP has a complex pathophysiology. First-generation antihistamines, such as diphenhydramine, hydroxyzine, and promethazine, are considered more suitable because they can cross the blood–brain barrier and exert an additional sedative effect. Hydroxyzine has additional antiserotonergic properties that may help reduce anxiety, a common cause of SD (Lavery et al., 2016). Second-generation antihistamines are thought to be less suited for NP because they lack sedative properties. Olopatadine (5 mg twice daily) has been reported to reduce NP in moderate-to-severe AD (Yamanaka et al., 2015). Although first-generation antihistamines are more effective, they should be used cautiously in elderly patients owing to their anticholinergic adverse effects, including arrhythmias, dizziness, and urinary retention.

#### Antidepressants

Mirtazapine, an atypical antidepressant (noradrelanine, serotonin, and histamine antagonist), is one of the most studied drugs in CP and NP refractory to conventional therapy. Mirtazapine can reduce NP associated with dermatological conditions such as AD (Khanna et al., 2019). The anxiolytic (serotonin antagonism) and sedative (histamine antagonism) properties of the drug are considered important in itch reduction. Several authors have recommended mirtazapine (7.5-15 mg at bedtime) as first-line treatment for NP because of its favorable safety profile. Mirtazapine can be also be used in combination with gamma-aminobutyric acid agonists for synergistic action (Lavery et al., 2016). Weight gain due to increased appetite is an adverse effect of mirtazapine.

Doxepin, a tricyclic antidepressant with antihistaminergic properties, has been tried in NP (25-150 mg at bedtime) with varying success. This agent has been effective in neuropathic and psychogenic itch (Yosipovitch and Samuel, 2008). Doxepin reduced sleep onset latency in adults with AD without any appreciable effect on NP in a small study (Savin et al., 1979). Amitryptilline, another tricyclic antidepressant (25-50 mg at bedtime), is frequently used for NP, and especially to treat psychogenic itching (Kuhn et al., 2017b). Selective serotonin reuptake inhibitors, especially paroxetine (25-50 mg/day), have been beneficial in NP and SD, particularly in the context of psychocutaneous disorders (Kuhn et al., 2017b).

#### Gamma-aminobutyric acid agonists

Gabapentin and pregabalin have been effectively used to treat NP. They have demonstrated efficacy in neuropathic and psychogenic pruritus, and their sedative action may play a beneficial role (Lavery et al., 2016; Pereira and Ständer, 2017).

#### Opioid receptor modulators

κ-opioid receptor agonists and κ-antagonists have demonstrated substantial efficacy in relieving intractable NP. Butorphanol, an agent having both properties, has been effective in NP associated with advanced age and chronic dermatoses, such as prurigo nodularis, at a dose 1 to 4 mg/day (inhaled; Dawn and Yosipovitch, 2006). Nalfurafine hydrochloride (2.5-5.0 μg/day) has been effective and safe in treating intractable NP in hemodialysis patients (Kozono et al., 2018).

#### Benzodiazepines

Benzodiazepines (BZDs) are among the most frequently prescribed medications to treat insomnia. These agents act by prolonging the N2 and reducing the N3 duration of sleep (Roux and Kryger, 2010). However, their efficacy in NP is debatable because a trial did not demonstrate any significant benefit of BZD in NP associated with AD (Ebata et al., 1998). Several adverse effects, such as tolerance, dependency, and exacerbation of symptoms on abrupt discontinuation, mandate the careful use of these agents. BZDs may increase daytime fatigue, somnolence, and psychomotor impairment due to their prolonged half-life (>8 hours).

#### Melatonin

Melatonin is an established treatment for delayed sleep phase disorder. Evidence for its effectiveness in insomnia is mixed (Bawany et al., 2020). In a small, well-designed, crossover trial in atopic children, melatonin (3 mg at bedtime) significantly reduced sleep-onset latency compared with placebo (Chang et al., 2016). However, the trial did not show any benefit on pruritus in the AD group compared with the placebo. Studies in adults are lacking.

#### Phototherapy

Narrowband ultraviolet B phototherapy has been effective in patients with CP (Yosipovitch and Bernhard, 2013). This modality may be tried in recalcitrant cases of NP, particularly those with generalized pruritus of unknown origin. Blue light has effectively reduced pruritus and SD in severe chronic AD (Becker et al., 2011). There are sporadic reports of effective use of psoralen plus ultraviolet light A therapy in prurigo nodularis (Kuhn et al., 2017b).

## Conclusions and future directions

NP is prevalent in a wide spectrum of skin disorders and causes impaired sleep quality. SD can result in poor physical and mental health, as well as daytime somnolence and tiredness leading to social adversities and decreased work productivity. Dermatologists should be aware of the common dermatologic associations of NP and impaired sleep quality. A detailed clinical history is crucial to detect the associated dermatologic condition. The value of elevated serum C-reactive protein levels as a biomarker of NP needs to be validated in large-scale studies.

The authors recommend a questionnaire-based assessment of sleep quality in all patients presenting with NP, and objective tests and referral to specialists should be considered in severe cases. Treatment should be approached in a patient-centric manner. Medications with dual antipruritic and soporific effects, including mirtazapine, gamma-aminobutyric acid agonists, and μ-opioid receptor agonists (e.g., butorphanol), may simultaneously target both itching and sleep impairment (Lavery et al., 2017). Emerging pruritus therapies, including neurokinin receptor 1 inhibitors (aprepitant, serlopitant, tradipitant), H4 antagonists, and mas-related G-protein coupled receptor blockers. appear promising in CP and possibly NP (Golpanian and Yosipovitch, 2020; Lonndahl et al., 2018). Large-scale studies are needed to evaluate the mechanisms of SD in dermatologic conditions and implement guidelines for effective management.

## Conflicts of interest

None.

## References

[bib0001] Abdel Latif O.M. (2017). Impact of severity of CSU on sleep, anxiety and depressive symptoms in adults. Eur Acad Res.

[bib0002] Allen R.P., Kosinski M., Hill-Zabala C.E., Calloway M.O. (2009). Psychometric evaluation and tests of validity of the medical outcomes study 12-item sleep scale (MOS sleep). Sleep Med.

[bib0003] Bae B.G., Oh S.H., Park C.O., Noh S., Noh J.Y., Kim K.R. (2012). Progressive muscle relaxation therapy for atopic dermatitis: Objective assessment of efficacy. Acta Derm Venereol.

[bib0004] Bawany F., Northcott C.A., Beck L.A., Pigeon W.R. (2020). Sleep disturbances and atopic dermatitis: Relationships, methods for assessment, and therapies. J Allergy Clin Immunol Pract.

[bib0005] Becker D., Langer E., Seemann M., Seemann G., Fell I., Saloga J. (2011). Clinical efficacy of blue light full body irradiation as treatment option for severe atopic dermatitis. PLoS One.

[bib0006] Bender B.G., Leung S.B., Leung D.Y. (2003). Actigraphy assessment of sleep disturbance in patients with atopic dermatitis: An objective life quality measure. J Allergy Clin Immunol.

[bib0007] Broomfield N.M., Espie C.A. (2005). Towards a valid, reliable measure of sleep effort. J Sleep Res.

[bib0008] Burns P.B., Rohrich R.J., Chung K.C. (2011). The levels of evidence and their role in evidence-based medicine. Plast Reconstr Surg.

[bib0009] Buysse D.J., Reynolds C.F., Monk T.H., Berman S.R., Kupfer D.J. (1989). Pittsburgh sleep quality index (PSQI): A new instrument for psychiatric practice and research. Psychiatry Res.

[bib0010] Carley D.W., Farabi S.S. (2016). Physiology of sleep. Diabetes Spectr.

[bib0011] Chang Y.S., Chiang B.L. (2016). Mechanism of sleep disturbance in children with atopic dermatitis and the role of the circadian rhythm and melatonin. Int J Mol Sci.

[bib0012] Chang Y.S., Lin M.H., Lee J.H., Lee P.L., Dai Y.S., Chu K.H. (2016). Melatonin supplementation for children with atopic dermatitis and sleep disturbance: A randomized clinical trial. JAMA Pediatr.

[bib0013] Cork M.J., Eckert L., Simpson E.L., Armstrong A., Barbarot S., Puig L. (2020). Dupilumab improves patient-reported symptoms of atopic dermatitis, symptoms of anxiety and depression, and health-related quality of life in moderate-to-severe atopic dermatitis: Analysis of pooled data from the randomized trials SOLO 1 and SOLO 2. J Dermatolog Treat.

[bib0014] Crönlein T., Langguth B., Popp R., Lukesch H., Pieh C., Hajak G. (2013). Regensburg Insomnia Scale (RIS): A new short rating scale for the assessment of psychological symptoms and sleep in insomnia; study design: Development and validation of a new short self-rating scale in a sample of 218 patients suffering from insomnia and 94 healthy controls. Health Qual Life Outcomes.

[bib0015] Dawn A.G., Yosipovitch G. (2006). Butorphanol for treatment of intractable pruritus. J Am Acad Dermatol.

[bib0016] Ebata T., Aizawa H., Kamide R., Niimura M. (1999). The characteristics of nocturnal scratching in adults with atopic dermatitis. Br J Dermatol.

[bib0017] Ebata T., Izumi H., Aizawa H., Kamide R., Niimura M. (1998). Effects of nitrazepam on nocturnal scratching in adults with atopic dermatitis: A double-blind placebo-controlled crossover study. Br J Dermatol.

[bib0018] Fargnoli M.C., Esposito M., Ferrucci S., Girolomoni G., Offidani A., Patrizi A., Dupilumab Italian National Access Program (Dup-INAP group) (2019). Real-life experience on effectiveness and safety of dupilumab in adult patients with moderate-to-severe atopic dermatitis. J Dermatolog Treat.

[bib0019] Folkard S. (2008). Do permanent night workers show circadian adjustment? A review based on the endogenous melatonin rhythm. Chronobiol Int.

[bib0020] Gimenéz-Arnau A.M., Spector S., Antonova E., Trzaskoma B., Rosén K., Omachi T.A. (2016). Improvement of sleep in patients with chronic idiopathic/spontaneous urticaria treated with omalizumab: Results of three randomized, double-blind, placebo-controlled studies. Clin Transl Allergy.

[bib0021] González-López G., Ceballos-Rodríguez R.M., González-López J.J., Feito Rodríguez M., Herranz-Pinto P. (2017). Efficacy and safety of wet wrap therapy for patients with atopic dermatitis: A systematic review and meta-analysis. Br J Dermatol.

[bib0022] Golpanian R.S., Yosipovitch G. (2020). Current and emerging systemic treatments targeting the neural system for chronic pruritus. Expert Opin Pharmacother.

[bib0023] Gupta M.A., Gupta A.K. (2013). Sleep-wake disorders and dermatology. Clin Dermatol.

[bib0024] Gwillim E.C., Janmohamed S.R., Yousaf M., Patel K.R., Silverberg J.I. (2020). The impact of prurigo nodularis on sleep disturbance and related impact: A systematic review. J Eur Acad Dermatol Venereol.

[bib0025] Haghayegh S., Khoshnevis S., Smolensky M.H., Diller K.R., Castriotta R.J. (2019). Before-bedtime passive body heating by warm shower or bath to improve sleep: A systematic review and meta-analysis. Sleep Med Rev.

[bib0026] Hawro T., Hawro M., Zalewska-Janowska A., Weller K., Metz M., Maurer M. (2020). Pruritus and sleep disturbances in patients with psoriasis. Arch Dermatol Res.

[bib0027] Henry A.L., Kyle S.D., Chisholm A., Griffiths C.E.M., Bundy C. (2017). A cross-sectional survey of the nature and correlates of sleep disturbance in people with psoriasis. Br J Dermatol.

[bib0028] Hoddes E., Zarcone V., Smythe H., Phillips R., Dement W.C. (1973). Quantification of sleepiness: A new approach. Psychophysiology.

[bib0029] Jafferany M., Davari M.E. (2019). Itch and psyche: Psychiatric aspects of pruritus. Int J Dermatol.

[bib0030] Jalili A., Lebwohl M., Stein Gold L., Andersen S.B., Jensen K.L., Pink A.E. (2019). Itch relief in patients with psoriasis: Effectiveness of calcipotriol plus betamethasone dipropionate foam. J Eur Acad Dermatol Venereol.

[bib0031] Jensen P., Zachariae C., Skov L., Zachariae R. (2018). Sleep disturbance in psoriasis: A case-controlled study. Br J Dermatol.

[bib0032] Jeon C., Yan D., Nakamura M., Sekhon S., Bhutani T., Berger T. (2017). Frequency and management of sleep disturbance in adults with atopic dermatitis: A systematic review. Dermatol Ther (Heidelb).

[bib0033] Johns M.W. (1991). A new method for measuring daytime sleepiness: The Epworth sleepiness scale. Sleep.

[bib0034] Kaaz K., Szepietowski J.C., Matusiak Ł. (2018). Influence of itch and pain on sleep quality in patients with hidradenitis suppurativa. Acta Derm Venereol.

[bib0035] Kaaz K., Szepietowski J.C., Matusiak Ł. (2019). Sleep quality among adult patients with chronic dermatoses. Postepy Dermatol Alergol.

[bib0036] Khanna R., Boozalis E., Belzberg M., Zampella J.G., Kwatra S.G. (2019). Mirtazapine for the treatment of chronic pruritus. Medicines (Basel).

[bib0037] Kim B., Jung H., Kim J., Lee J., Kim O. (2020). Depressive symptoms and sleep disturbance in female nurses with atopic dermatitis: The Korea Nurses' Health Study. Int J Environ Res Public Health.

[bib0038] Kimball A.B., Edson-Heredia E., Zhu B., Guo J., Maeda-Chubachi T., Shen W. (2016). Understanding the relationship between pruritus severity and work productivity in patients with moderate-to-severe psoriasis: Sleep problems are a mediating factor. J Drugs Dermatol.

[bib0039] Koca R., Altin R., Konuk N., Altinyazar H.C., Kart L. (2006). Sleep disturbance in patients with lichen simplex chronicus and its relationship to nocturnal scratching: A case control study. South Med J.

[bib0040] Kontochristopoulos G., Kouris A., Chantzaras A., Petridis A., Yfantopoulos J. (2016). Improvement of health-related quality of life and adherence to treatment with calcipotriol-betamethasone dipropionate gel in patients with psoriasis vulgaris. An Bras Dermatol.

[bib0041] Kong T.S., Han T.Y., Lee J.H., Son S.J. (2016). Correlation between severity of atopic dermatitis and sleep quality in children and adults. Ann Dermatol.

[bib0042] Kozono H., Yoshitani H., Nakano R. (2018). Post-marketing surveillance study of the safety and efficacy of nalfurafine hydrochloride (Remitch® capsules 2.5 μg) in 3762 hemodialysis patients with intractable pruritus. Int J Nephrol Renovasc Dis.

[bib0043] Kuhn H., Mennella C., Magid M., Stamu-O'Brien C., Kroumpouzos G. (2017). Psychocutaneous disease: Clinical perspectives. J Am Acad Dermatol.

[bib0044] Kuhn H., Mennella C., Magid M., Stamu-O'Brien C., Kroumpouzos G. (2017). Psychocutaneous disease: Pharmacotherapy and psychotherapy. J Am Acad Dermatol.

[bib0045] Lavery M.J., Stull C., Kinney M.O., Yosipovitch G. (2016). Nocturnal pruritus: The battle for a peaceful night's sleep. Int J Mol Sci.

[bib0046] Lavery M.J., Stull C., Nattkemper L.A., Sanders K.M., Lee H., Sahu S. (2017). Nocturnal pruritus: Prevalence, characteristics, and impact on ItchyQoL in a chronic itch population. Acta Derm Venereol.

[bib0047] Lei D., Yousaf M., Janmohamed S.R., Vakharia P.P., Chopra R., Chavda R. (2020). Validation of four single-item patient-reported assessments of sleep in adult atopic dermatitis patients. Ann Allergy Asthma Immunol.

[bib0048] Lei D.K., Yousaf M., Janmohamed S.R., Vakharia P.P., Chopra R., Sacotte R. (2020). Validation of patient-reported outcomes information system sleep disturbance and sleep-related impairment in adults with atopic dermatitis. Br J Dermatol.

[bib0049] Li J.C., Fishbein A., Singam V., Patel K.R., Zee P.C., Attarian H. (2018). Sleep disturbance and sleep-related impairment in adults with atopic dermatitis: A cross-sectional study. Dermatitis.

[bib0050] Ljosaa T.M., Mork C., Stubhaug A., Moum T., Wahl A.K. (2012). Skin pain and skin discomfort is associated with quality of life in patients with psoriasis. J Eur Acad Dermatol Venereol.

[bib0051] Lönndahl L., Holst M., Bradley M., Killasli H., Heilborn J., Hall M.A. (2018). Substance P antagonist aprepitant shows no additive effect compared with standardized topical treatment alone in patients with atopic dermatitis. Acta Derm Venereol.

[bib0052] Monti J.M., Vignale R., Monti D. (1989). Sleep and nighttime pruritus in children with atopic dermatitis. Sleep.

[bib0053] Mrowietz U., Chouela E.N., Mallbris L., Stefanidis D., Marino V., Pedersen R. (2015). Pruritus and quality of life in moderate-to-severe plaque psoriasis: Post hoc explorative analysis from the PRISTINE study. J Eur Acad Dermatol Venereol.

[bib0054] Parish J.M. (2009). Sleep-related problems in common medical conditions. Chest.

[bib0055] Patel T., Ishiuji Y., Yosipovitch G. (2007). Nocturnal itch: Why do we itch at night?. Acta Derm Venereol.

[bib0056] Patel S.P., Khanna R., Choi J., Williams K.A., Roh Y.S., Hong M.S. (2021). Sleep disturbance in adults with chronic pruritic dermatoses is associated with increased C-reactive protein levels. J Am Acad Dermatol.

[bib0057] Pereira M.P., Ständer S. (2017). Chronic pruritus: Current and emerging treatment options. Drugs.

[bib0058] Potter G.D., Skene D.J., Arendt J., Cade J.E., Grant P.J., Hardie L.J. (2016). Circadian rhythm and sleep disruption: Causes, metabolic consequences, and countermeasures. Endocr Rev.

[bib0059] Roux F.J., Kryger M.H. (2010). Medication effects on sleep. Clin Chest Med.

[bib0060] Savin J.A., Paterson W.D., Adam K., Oswald I. (1979). Effects of trimeprazine and trimipramine on nocturnal scratching in patients with atopic eczema. Arch Dermatol.

[bib0061] Schut C., Mollanazar N.K., Kupfer J., Gieler U., Yosipovitch G. (2016). Psychological interventions in the treatment of chronic itch. Acta Derm Venereol.

[bib0062] Shen M., Xiao Y., Su J., Zhao S., Li J., Tao J. (2020). Prevalence and patient-reported outcomes of noncommunicable skin diseases among college students in China. JAAD Int.

[bib0063] Shutty B.G., West C., Huang K.E., Landis E., Dabade T., Browder B. (2013). Sleep disturbances in psoriasis. Dermatol Online J.

[bib0064] Silverberg J.I., Chiesa Fuxench Z.C., Gelfand J.M., Margolis D.J., Boguniewicz M., Fonacier L. (2018). Content and construct validity, predictors, and distribution of self-reported atopic dermatitis severity in US adults. Ann Allergy Asthma Immunol.

[bib0065] Silverberg J.I., Garg N.K., Paller A.S., Fishbein A.B., Zee P.C. (2015). Sleep disturbances in adults with eczema are associated with impaired overall health: A U.S. population-based study. J Invest Dermatol.

[bib0066] Silverberg J.I., Gelfand J.M., Margolis D.J., Boguniewicz M., Fonacier L., Grayson M.H. (2018). Patient burden and quality of life in atopic dermatitis in U.S. adults: A population-based cross-sectional study. Ann Allergy Asthma Immunol.

[bib0067] Silverberg J.I., Lei D., Yousaf M., Janmohamed S.R., Vakharia P.P., Chopra R. (2020). Association of itch triggers with atopic dermatitis severity and course in adults. Ann Allergy Asthma Immunol.

[bib0068] Simpson E.L., Bieber T., Eckert L., Wu R., Ardeleanu M., Graham N.M. (2016). Patient burden of moderate to severe atopic dermatitis (AD): Insights from a phase 2b clinical trial of dupilumab in adults. J Am Acad Dermatol.

[bib0069] Simpson E.L., Guttman-Yassky E., Margolis D.J., Feldman S.R., Qureshi A., Hata T. (2018). Association of inadequately controlled disease and disease severity with patient-reported disease burden in adults with atopic dermatitis. JAMA Dermatol.

[bib0070] Smith M.P., Ly K., Thibodeaux Q., Weerasinghe T., Beck K., Shankle L. (2019). Factors influencing sleep difficulty and sleep quantity in the Citizen Pscientist psoriatic cohort. Dermatol Ther (Heidelb).

[bib0071] Soldatos C.R., Dikeos D.G., Paparrigopoulos T.J. (2000). Athens insomnia scale: Validation of an instrument based on ICD-10 criteria. J Psychosom Res.

[bib0072] Stinco G., Trevisan G., Piccirillo F., Di Meo N., Nan K., Deroma L. (2013). Psoriasis vulgaris does not adversely influence the quality of sleep. G Ital Dermatol Venereol.

[bib0073] Strober B.E., Sobell J.M., Duffin K.C., Bao Y., Guérin A., Yang H. (2012). Sleep quality and other patient-reported outcomes improve after patients with psoriasis with suboptimal response to other systemic therapies are switched to adalimumab: Results from PROGRESS, an open-label Phase IIIB trial. Br J Dermatol.

[bib0074] Suilmann T., Zeidler C., Osada N., Riepe C., Ständer S. (2018). Usability of validated sleep-assessment questionnaires in patients with chronic pruritus: An interview-based study. Acta Derm Venereol.

[bib0075] Tas B., Kabeloglu V., Soysal A., Atakli D. (2020). Sleep quality in psoriasis patients and its relations with possible affecting factors. Sisli Etfal Hastan Tip Bul.

[bib0076] Tivoli Y.A., Rubenstein R.M. (2009). Pruritus: An updated look at an old problem. J Clin Aesthet Dermatol.

[bib0077] Togeiro S.M., Smith A.K. (2005). Diagnostics methods for sleep disorders. Braz J Psychiatry.

[bib0078] Tsianakas A., Luger T.A., Radin A. (2018). Dupilumab treatment improves quality of life in adult patients with moderate-to-severe atopic dermatitis: Results from a randomized, placebo-controlled clinical trial. Br J Dermatol.

[bib0079] Vakharia P.P., Chopra R., Sacotte R., Patel K.R., Singam V., Patel N. (2017). Burden of skin pain in atopic dermatitis. Ann Allergy Asthma Immunol.

[bib0080] Vossen A.R.J.V., Schoenmakers A., van Straalen K.R., Prens E.P., van der Zee H.H. (2017). Assessing pruritus in hidradenitis suppurativa: A cross-sectional study. Am J Clin Dermatol.

[bib0081] Wei W., Anderson P., Gadkari A., Blackburn S., Moon R., Piercy J. (2018). Extent and consequences of inadequate disease control among adults with a history of moderate to severe atopic dermatitis. J Dermatol.

[bib0082] Worth C., Heukelbach J., Fengler G., Walter B., Liesenfeld O., Hengge U. (2012). Acute morbidity associated with scabies and other ectoparasitoses rapidly improves after treatment with ivermectin. Pediatr Dermatol.

[bib0083] Wu Y., Mills D., Bala M. (2008). Psoriasis: Cardiovascular risk factors and other disease comorbidities. J Drugs Dermatol.

[bib0084] Yamanaka K., Motomura E., Noro Y., Umeda K., Morikawa T., Umeda-Togami K. (2015). Olopatadine, a non-sedating H1 antihistamine, decreases the nocturnal scratching without affecting sleep quality in atopic dermatitis. Exp Dermatol.

[bib0085] Yano C., Saeki H., Ishiji T., Ishiuji Y., Sato J., Tofuku Y. (2013). Impact of disease severity on sleep quality in Japanese patients with atopic dermatitis. J Dermatol Sci.

[bib0086] Yosipovitch G., Bernhard J.D. (2013). Chronic pruritus. N Engl J Med.

[bib0087] Yosipovitch G., Samuel L.S. (2008). Neuropathic and psychogenic itch. Dermatol Ther.

[bib0088] Yu S.H., Attarian H., Zee P., Silverberg J.I. (2016). Burden of sleep and fatigue in U.S. adults with atopic dermatitis. Dermatitis.

[bib0089] Zachariae R., Lei U., Haedersdal M., Zachariae C. (2012). Itch severity and quality of life in patients with pruritus: Preliminary validity of a Danish adaptation of the itch severity scale. Acta Derm Venerol.

